# ^99m^Tc-Sn-colloid SPECT/CT in thoracic splenosis after esophageal cancer surgery

**DOI:** 10.22038/AOJNMB.2023.73907.1515

**Published:** 2024

**Authors:** Kishin Tokuyama, Yusuke Inoue, Keiji Matsunaga, Yasunori Hamaguchi, Saori Sekimoto

**Affiliations:** 1 Department of Radiology, Kitasato University Hospital, Sagamihara, Kanagawa, Japan; 2 Department of Radiology, Fuchinobe General Hospital, Sagamihara, Kanagawa, Japan

**Keywords:** ^99m^Tc-labeled colloid SPECT/CT, Thoracic splenosis, Esophageal cancer, Gastric tube reconstruction

## Abstract

Splenosis occurs as a result of autotransplantation of splenic tissue following splenic injury or splenectomy. A 56-year-old man with esophageal cancer underwent thoracoscopic-assisted subtotal esophagectomy accompanied by three-field lymph node dissection, and retrosternal gastric tube reconstruction. The spleen was injured during the surgery and was removed. A retrosternal nodule of 12 mm in diameter was detected near the reconstructed gastric tube on computed tomography (CT) performed 3 years and 6 months postoperatively.

Retrospectively, the nodule was observed in the same area on early postoperative CT and gradually increased in size. No accessory spleen was identified on the preoperative CT. Splenosis was suspected, and ^99m^Tc-Sn-colloid single photon emission computed tomography (SPECT)/CT was performed. It revealed intense uptake in the retrosternal nodule, consistent with the diagnosis of thoracic splenosis. Subsequently, the patient has been under observation without treatment. ^99m^Tc-labeled colloid SPECT/CT allowed confident diagnosis of thoracic splenosis following esophageal cancer surgery. This examination is considered valuable for the evaluation of ectopic splenic tissue.

## Introduction

 Splenosis occurs as a result of autotransplantation of splenic tissue following splenic injury or splenectomy, while accessory spleen is a congenital organ that arises from incomplete fusion of the splenic primordium (1, 2). The diagnosis of such ectopic splenic tissue involves various examinations, including ultrasonography, computed tomography (CT), magnetic resonance (MR) imaging, nuclear medicine imaging, and surgical biopsy (3, 4). We report a patient in whom ^99m^Tc-Sn-colloid single photon emission computed tomography/ computed tomography (SPECT/CT) allowed confident diagnosis of thoracic splenosis following gastric tube reconstruction for esophageal cancer.

## Case Report

 A 56-year-old male patient presented with chest discomfort during meals and was diagnosed with middle thoracic esophageal cancer (cT3N1M0, cStage IIIb). After neoadjuvant chemotherapy, he underwent thoracoscopic-assisted subtotal esophagectomy accompanied by three-field lymph node dissection and retrosternal gastric tube reconstruction. During the preparation of the gastric tube, the splenic artery was injured, leading to conversion from laparoscopic surgery to open surgery for hemostasis. Although the source of bleeding was repaired, ischemic findings of the spleen were observed, and splenectomy was performed simultaneously. 

 The postoperative diagnosis was middle thoracic esophageal cancer (pTisN0M0, pStage 0).

**Figure 1:**
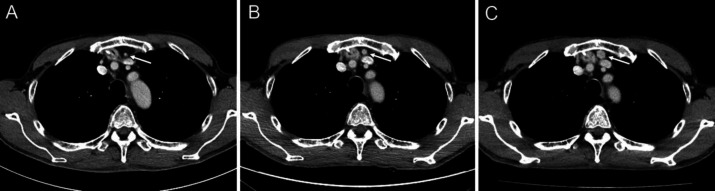
Serial postoperative contrast-enhanced CT of the chest at 3 months (**A**), a year and 3 months (**B**), and 2 years and 3 months (**C**) postoperatively. A small nodule is identified near the reconstructed gastric tube, showing gradual enlargement (**arrows**)

 During a follow-up, plain CT performed 3 years and 6 months postoperatively demonstrated a retrosternal nodule of 12 mm in diameter near the reconstructed gastric tube. The nodule had smooth margins, a round shape, and homogeneous density. Upon retrospective review of postoperative contrast-enhanced CT, a corresponding nodule showing gradual enlargement was identified (Figure 1). The nodule showed homogeneously high density, suggesting homogeneous contrast enhancement. 

**Figure 2:**
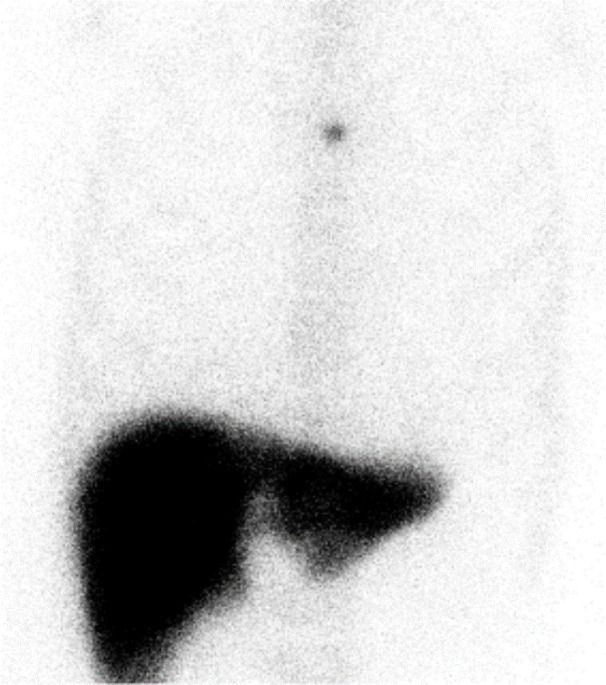
The anterior planar image of ^99m^Tc-Sn-colloid scan at 3 years and 6 months postoperatively showed focal uptake in the center of the upper chest. Radioactivity in the native spleen is absent

**Figure 3:**
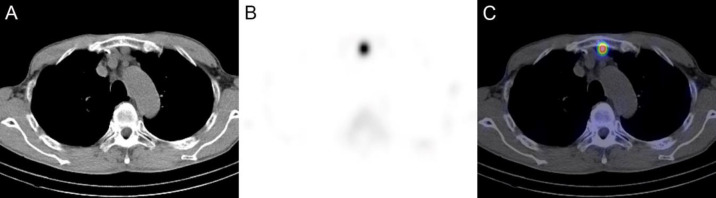
^99m^Tc-Sn-colloid SPECT/CT (**A**, plain CT; **B**, SPECT; C, fused SPECT/CT). Intense accumulation was observed in the anterior mediastinum, and the fused SPECT/CT images demonstrated that the accumulation corresponded to the nodule of interest

 Based on the CT appearances and slow enlargement, ectopic splenic tissue was suspected rather than cancer recurrence or metastasis. Because no similar nodules were observed in the abdomen or anterior mediastinum on preoperative CT, splenosis related to gastric tube reconstruction was suspected. To confirm the diagnosis, ^99m^Tc-Sn-colloid SPECT/CT was performed. ^99m^Tc-Sn-colloid (115 MBq) was administered intravenously, and 15 minutes later, planar images of the chest and abdomen and SPECT/CT images of the chest were acquired using a SPECT/CT scanner (Symbia T2; Siemens, Erlangen, Germany) equipped with a low-energy high-resolution collimator. Intense accumulation was observed in the chest (Figure 2), and the fused SPECT/CT images confirmed that the accumulation corresponded to the nodule of interest (Figure 3). The diagnosis of ectopic splenic tissue was established confidently, and no treatment was deemed necessary. Subsequent contrast-enhanced CT examination at 5 years post-operatively revealed a mild further increase in the size of the nodule to 16 mm, but no recurrence or metastasis of esophageal cancer was detected.

## Discussion

^99m^Tc-labeled colloid, including ^99m^Tc-Sn-colloid, is taken up physiologically by the reticuloendothelial system and accumulates in the liver, spleen, and bone marrow (5). While ^99m^Tc-heat-damaged red blood cells exhibit higher specificity to the spleen, ^99m^Tc-labeled colloid is more convenient, and its usefulness for the evaluation of the morphology and funciton of the spleen including the detection of ectopic splenic tissues is well accepted (6). The application of ^99m^Tc-labeled colloid SPECT for the diagnosis of post-traumatic thoracic splenosis has been reported (7). Although planar imaging may be performed in ^99m^Tc-labeled colloid scintigraphy, SPECT allows three-dimensional localization. Furthermore, the use of ^99m^Tc-labeled colloid SPECT/CT has been reported for the diagnosis of intrapancreatic accessory spleen (8). In SPECT/CT, SPECT offers functional or tissue-specific information and CT permits accurate localization, thereby providing high diagnostic capability (9). In this patient, the focal accumulation in the anterior mediastinum was delineated with prominently high contrast to the surrounding tissues through selective accumulation of ^99m^Tc-Sn-colloid by splenic tissues, confirming the diagnosis of ectopic splenic tissue. Additionally, the fusion images confirmed that this accumulation corresponded to the nodule that had been detected in the preceding CT.

 Superparamagnetic iron oxide (SPIO) MR imaging has also been performed to diagnose ectopic splenic tissue (10, 11). SPIO is taken up by reticuloendothelial cells, causing shortening of T2 and T2* relaxation times, resulting in signal decrease in T2-weighted and T2*-weighted images. SPIO MR imaging examination is considered useful for the evaluation of reticuloendothelial system due to its high spatial and tissue resolution. However, in the patient presented here, the lesion was located in the anterior mediastinum, and thus respiratory motion artifacts on MR imaging were concerned. 

^99m^Tc-Sn-colloid SPECT/CT was selected instead of SPIO MR imaging, and confident diagnosis was established. Because of the high confidence, surgical biopsy was omitted. Lack of pathological diagnosis may be a limitation of our report; however, the absence of any metastasis or recurrence, even during an additional follow-up of 1 year and 6 months, further supports the diagnosis of splenosis rather than metastasis.

 Typically, splenosis occurs in the abdomen after splenic injury due to trauma or splenectomy (2). Although atypical, splenosis may occur in the thoracic region. In a previous report of 62 patients with thoracic splenosis, the etiology was gunshot wounds in 31 patients, motor vehicle accidents in 18, road traffic accidents in 8, stab wounds in 1, and other traumas in 6, with trauma being involved in all patients (3). It is speculated that trauma damages the spleen and diaphragm and that detached splenic tissue moves through the diaphragm into the mediastinum, followed by enlargement of ectopic splenic tissue under the absence of the native spleen. In the patient presented here, it is assumed that the spleen was damaged during the surgery, and the detached splenic tissue adhered to the tissues around the stomach, followed by its enlargement due to the post-splenectomy status. Although an accessory spleen in the abdomen was not identified even in retrospective evaluation of preoperative CT, it is also conceivable that a pre-existing accessory spleen migrated into the mediastinum during stomach tube reconstruction.

## Conclusion

 Thoracic splenosis may occur after stomach tube reconstruction and splenectomy in patients with esophageal cancer. ^99m^Tc-labeled-colloid SPECT/CT is considered to offer high confidence in diagnosing ectopic splenic tissue through excellent contrast to surrounding tissues and ability of precise localization.
